# Peripheral Vγ9Vδ2 T Cells Are a Novel Reservoir of Latent HIV Infection

**DOI:** 10.1371/journal.ppat.1005201

**Published:** 2015-10-16

**Authors:** Natalia Soriano-Sarabia, Nancie M. Archin, Rosalie Bateson, Noelle P. Dahl, Amanda M. Crooks, JoAnn D. Kuruc, Carolina Garrido, David M. Margolis

**Affiliations:** 1 Department of Medicine, University of North Carolina at Chapel Hill, Chapel Hill, North Carolina, United States of America; 2 Department of Microbiology and Immunology, University of North Carolina at Chapel Hill, Chapel Hill, North Carolina, United States of America; 3 Department of Epidemiology, University of North Carolina at Chapel Hill, Chapel Hill, North Carolina, United States of America; Emory University, UNITED STATES

## Abstract

Eradication of HIV infection will require the identification of all cellular reservoirs that harbor latent infection. Despite low or lack of CD4 receptor expression on Vδ2 T cells, infection of these cells has previously been reported. We found that upregulation of the CD4 receptor may render primary Vδ2 cells target for HIV infection *in vitro* and we propose that HIV-induced immune activation may allow infection of γδ T cells *in vivo*. We assessed the presence of latent HIV infection by measurements of DNA and outgrowth assays within Vδ2 cells in 18 aviremic patients on long-standing antiretroviral therapy. In 14 patients we recovered latent but replication-competent HIV from highly purified Vδ2 cells demonstrating that peripheral Vδ2 T cells are a previously unrecognized reservoir in which latent HIV infection is unexpectedly frequent.

## Author Summary

Antiretroviral therapy (ART) has led to a decreased HIV-related morbidity and mortality across the world. While successful ART restores health, it does not cure infection as latent HIV-1 remains integrated within different cell populations, unaffected by ART. To date resting memory CD4^+^ T cells are the best-characterized cellular reservoir. However, eradication of HIV-1 infection requires the description of all latent cellular reservoirs harboring replication-competent HIV-1. We describe the discovery of an unexpected cellular reservoir within γδ T lymphocytes. This novel reservoir must be considered as strategies to clear latent HIV are developed and tested.

## Introduction

The infecting HIV genome integrates into host chromatin where, transcriptionally silent and unaffected by antiretroviral therapy (ART), it represents a major challenge towards efforts to eradicate infection [1]. Virologic latency is defined as durable, quiescent infection from which replication-competent HIV can emerge after cell activation. To date, resting memory CD4^+^ T lymphocytes are the major cell type in which latency has been documented *in vivo* [2–5]. However, efforts to eradicate HIV infection require the identification of all potential cellular reservoirs and therefore, while conventional αβ T cells, which include resting memory CD4^+^ T cells, constitute the major subpopulation of T lymphocytes, the γδ T cell population merits study as a potentially important site of latent infection.

In the absence of pathological conditions such as infection, γδ T cells represent between 2 and 10% of total circulating CD3^+^ T lymphocytes [6]. Among peripheral CD3^+^ γδ T cells, those expressing a TCR formed by the Vγ9 and Vδ2 variable regions (hereafter referred to as Vδ2 cells) constitute up to 90% of γδ T cells [7]. These Vδ2 cells specifically recognize non-peptidic phosphorylated metabolites of isoprenoid biosynthesis, such as the potent activator (E)-4-hydroxy-3-methyl-but-2-enyl pyrophosphate (HMBPP), present in most pathogenic bacteria [8,9], or isopentenyl pyrophosphate (IPP), produced also by the human mevalonate biosynthesis pathway [10], but are not recognized by conventional αβ T cells. *In vitro*, both compounds have the same effect on γδ T cells, activating them. In adults, most Vδ2 cells are memory cells that can be further classified according to expression of CD45RO and CD27 surface markers [11–13].

During HIV-1 infection, the peripheral blood Vδ2 cell subset is depleted while Vδ1 cells are expanded, leading to an inversion of the Vδ2/Vδ1 ratio [14,15]. An indirect mechanism involving CCR5/α4β7 signalling was hypothesized to explain the specific depletion of Vδ2 cells [16]. However, an additional mechanism may be the direct infection of these cells as productive HIV infection of γδ T cells within peripheral blood mononuclear cells (PBMC) [17] as well as infection of γδ T cell clones by the CXCR4-tropic laboratory clone HIV_LAI_ [18] has been reported. Similarly, SIV can infect both Vδ1 and Vδ2 cells, albeit infrequently [19]. Despite the documented capacity of HIV to infect γδ T cells, the potential of γδ T cells to serve as a persistent reservoir of infection has not been studied. Moreover, the memory phenotype of Vδ2 cells suggests that these cells could play a role as durable *in vivo* reservoirs of HIV infection. Using a viral outgrowth assay to detect latent but replication-competent HIV [20,21], complemented by measures of HIV DNA, we demonstrate for the first time that peripheral Vδ2 cells in ART-treated patients with complete suppression of HIV plasma viremia harbour latent HIV that can replicate following *ex vivo* induction. We report the discovery of a new reservoir of HIV within peripheral Vδ2 cells, and suggest that infection in this population may be founded by immune activation that transiently upregulates the CD4 receptor on Vδ2 cells.

## Results

### Patients’ characteristics

To study the role of Vδ2 cells as reservoirs of persistent, latent HIV infection, 18 HIV-infected male volunteers, who initiated ART in acute HIV infection (AHI; n = 9) or in chronic HIV infection (CHI; n = 9) and received stable ART for a median of 3.4 years [range 1.9–9.5] were studied. A comparison between AHI and CHI-treated patients’ characteristics at the time of study showed that CHI patients had, as expected, a statistically significant lower nadir CD4 count (p = 0.017) and a significantly longer time on ART (p = 0.004). Median CD8^+^ T cell count was lower and pre-therapy plasma HIV RNA was higher in the AHI patients although these differences did not achieve statistical significance (**Table 1**).

**Table 1:** Patients’ characteristics at study entry: Comparison between patients treated in acute HIV infection (AHI) and in chronic HIV infection (CHI).

	Patient	CD4 nadir (cells/mm^3^)	VL (Log_10_ copies/mL)†	CD4 count (cells/mm^3^)	CD8 count (cells/mm^3^)	Time on ART (years)	Time suppressed (years)
**AHI**	**A.1**	840	6.86	870	552	2.44	2.22
	**A.2**	600	5.27	599	684	2.15	1.55
	**A.3**	371	5.19	629	398	3.38	3.22
	**A.4**	137	6.24	776	592	4.04	3.12
	**A.5**	277	7.93	615	464	6.46	3.99
	**A.6**	520	4.36	698	470	0.98	0.77
	**A.7**	606	6.06	864	940	1.38	1.36
	**A.8**	474	7.00	967	684	0.94	0.5
	**A.9**	409	4.40	931	1123	1.42	0.7
	**Median**	474	6.06	776	592	2.14	1.55
**CHI**	**C.1**	n/a	n/a	711	834	8.48	3.31
	**C.2**	81	5.58	772	464	12.52	2.76
	**C.3**	195	4.86	1027	652	26.93	4.29
	**C.4**	322	5.57	830	1014	3.01	2.72
	**C.5**	602	5.88	1120	1013	5.31	4.98
	**C.6**	180	5.24	727	1036	20.12	11.83
	**C.7**	232	4.80	495	775	3.41	2.87
	**C.8**	243	5.39	608	925	19.99	3.97
	**C.9**	338	5.27	1145	1117	3.14	0.07
	**Median**	238	5.32	772	925	8.48	3.31
**p** *		**0.043**	0.38	0.69	0.09	**0.004**	0.058

† VL = Viral Load before suppression

*Mann-Whitney *U*-test

n/a = not available.

### Purity of Vδ2 cells

**Fig 1:**
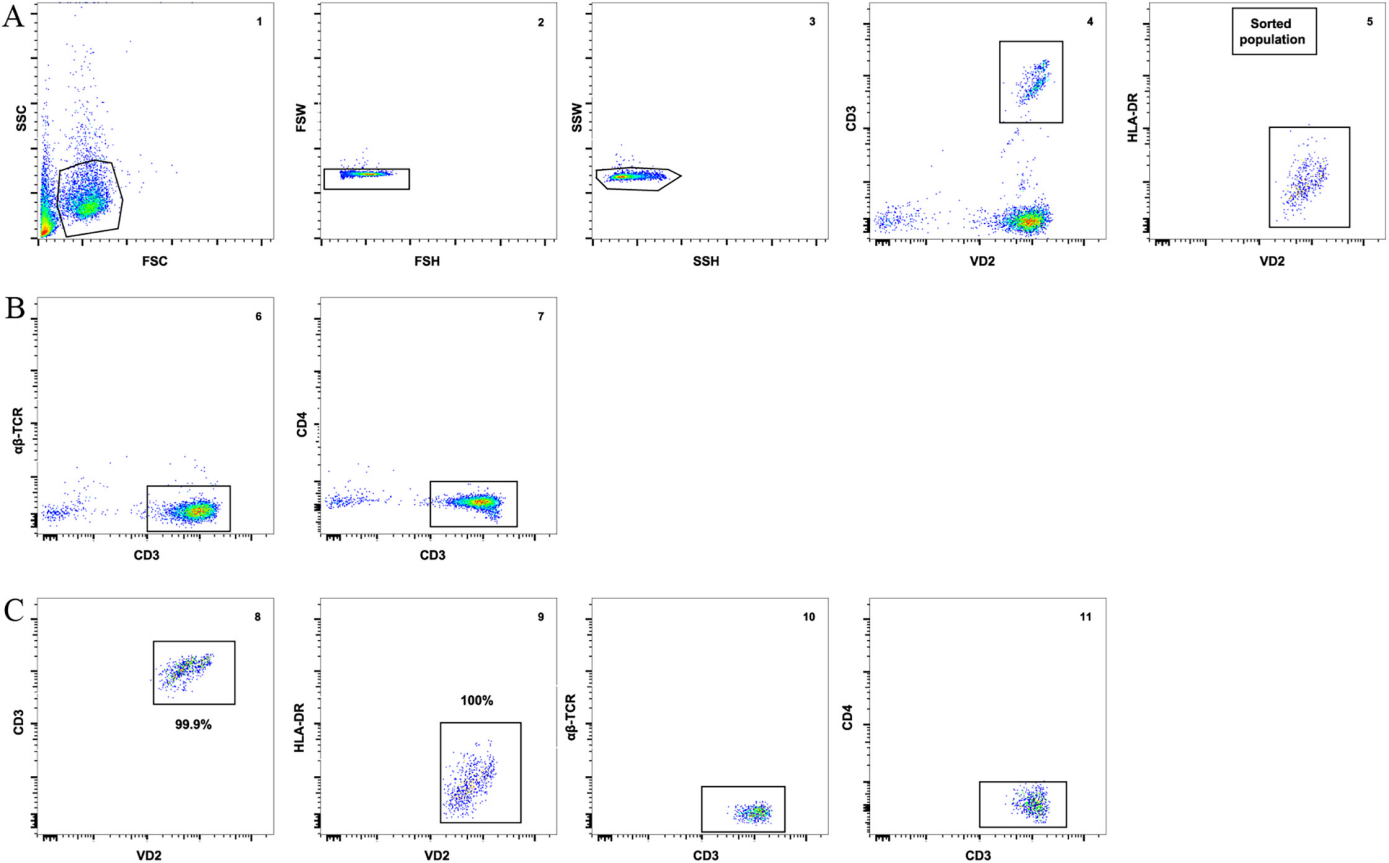
Vδ2 T cell sorting strategy and purity. **A)** An example of the gating strategy to sort pre-enriched Vδ2 cells (panel 1) using a 2-step doublet discrimination (panels 2 and 3), showing that CD3^+^ Vδ2 cells (panel 4) lack expression of HLA-DR (panel 5). **B)** Dot plot examples showing lack of αβ-TCR (panel 6) and CD4 (panel 7) expression in the presort sample. **C)** Purity of the sorted cell population. After the sort an aliquot of sorted cells was acquired and analyzed. Plots show high purity of CD3^+^ Vδ2 cells (panel 8) and lack of HLA-DR expression (panel 9). Post-sort analysis shows lack of αβ-TCR and CD4 (panels 10 and 11).

To ensure that other contaminating cells did not contribute to the recovery of HIV from isolated Vδ2 cells, we incubated freshly isolated patients’ PBMC with raltegravir and abacavir for 24 hours to avoid the possibility that *de novo* integration events could occur *ex vivo* after cell donation. γδ T cells were then enriched from PBMC using magnetic immunoaffinity beads, and non-activated (HLA-DR^-^) Vδ2 cells were further purified by FACS-sorting (Fig 1A and 1B). This process excluded αβTCR^+^ cells (classical CD4^+^ T cells) from pre-sort samples (Fig 1C), as detailed in Materials and Methods. To further confirm that Vδ2 cells were not already activated, aliquots of isolated Vδ2 cells were cultured in 5U/mL IL-2 prior to the addition of target cells in the viral outgrowth assay. HIV p24 measurements from these cultures were uniformly negative.

### Vδ2 cells contain proviral HIV DNA

**Fig 2:**
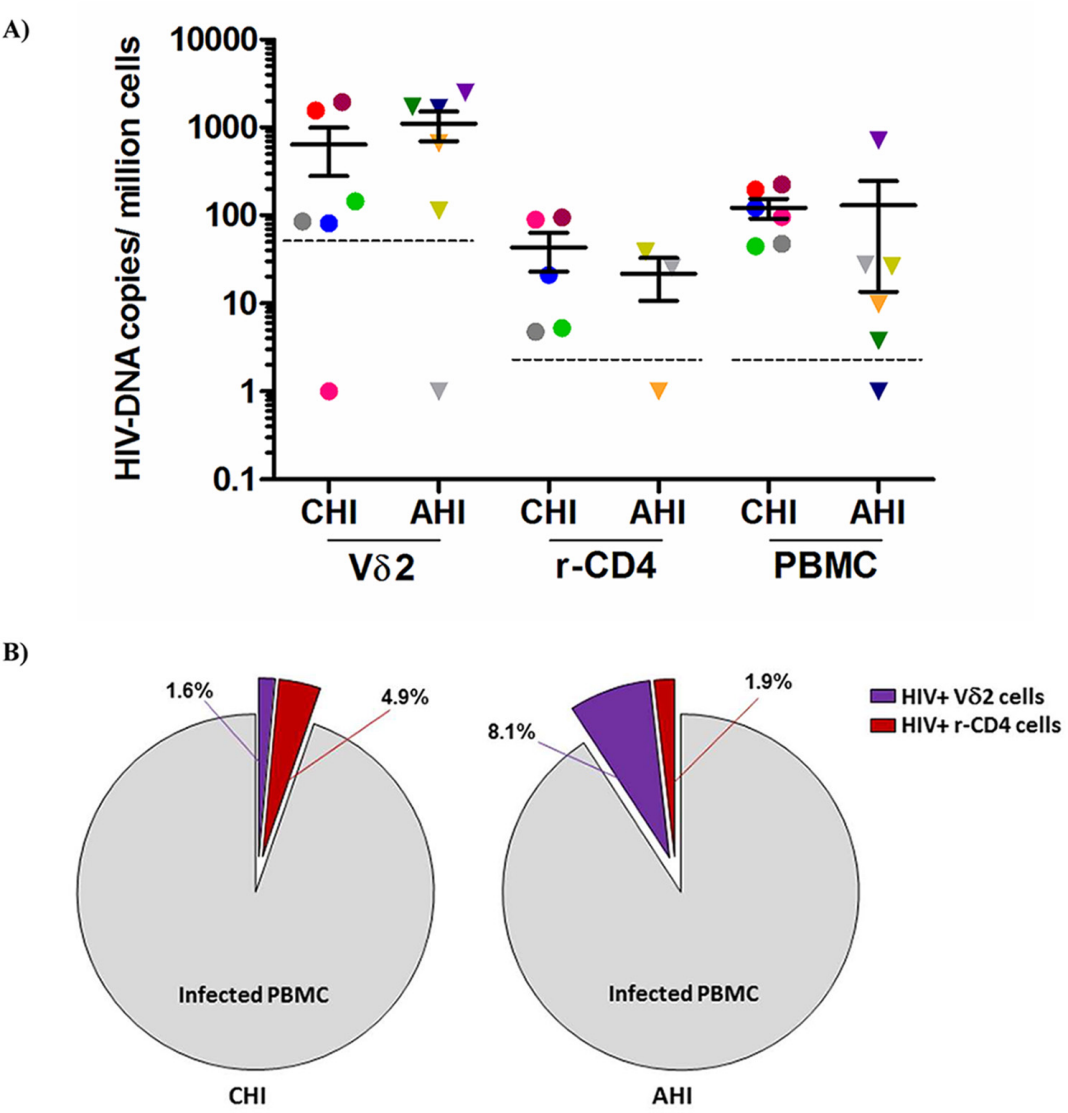
Quantification of total HIV DNA levels. **A)** Total *pol* HIV copies were quantified by ddPCR within Vδ2 cells (n = 12), total resting CD4^+^ T cells (r-CD4) (n = 8) and unfractionated PBMC (n = 12) from HIV-1 suppressed patients treated in the acute HIV infection (AHI) or in the chronic HIV infection (CHI). Limit of quantitation (LOQ) was 50.6 copies/ 10^6^ Vδ2 cells and 5.1 copies/ 10^6^ r-CD4 cells and PBMC, and is depicted with a dotted line. Each color represents one patient. **B)** Pie charts reflecting the contribution of Vδ2 cells (purple) and r-CD4 cells (red) to the total HIV DNA^+^ PBMC in CHI patients (left pie) and AHI patients (right pie).

Total HIV DNA levels were then quantified in isolated Vδ2 cells, unfractionated PBMC and total resting CD4^+^ T (r-CD4) cells, when available (Fig 2A) in patients treated in AHI and CHI. As previously published in studies of other cell populations [22], DNA levels varied widely but interestingly, Vδ2 cells showed the highest level of *pol* HIV DNA copies per 10^6^ Vδ2 cells (mean of 873.6 HIV copies/10^6^ cells). Due to the low number of Vδ2 cells available for analysis the limit of quantitation of Vδ2 cells was 50.6 copies/10^6^ cells, and 5.1 copies/10^6^ cells for the other cell populations, where more cells could be analyzed. HIV DNA levels within Vδ2 cells were not statistically different between AHI and CHI-treated patients (p = 0.37). Within PBMC and r-CD4 cells HIV DNA levels were higher in CHI patients than in AHI patients, although this difference did not achieve statistical significance (p = 0.06 for PBMC and p = 0.65 for resting CD4^+^ T cells). We recovered an average of 638.6 HIV DNA copies/million γδ T cells from CHI patients, and an average of 1108.7 copies/ million from AHI patient. Conversely, we recovered an average of 30.3 HIV DNA copies/ million rCD4 cells from CHI patients and an average of 21.4 copies/ 10^6^ rCD4 cells from AHI patients. We calculated the contribution of HIV DNA in Vδ2 cells and r-CD4 cells to the total HIV-DNA^+^ PBMC (Fig 2B) as follows: First, we calculated the total HIV DNA copies in each cell population by multiplying the average HIV copy number per million cells to the percentage of Vδ2 or r-CD4 cells present in total PBMC. Then, this total HIV copy number was divided by the total HIV copy number in PBMC to obtain the proportion of HIV corresponding to Vδ2or r-CD4 populations. Vδ2 cells contributed 1.6% and 8.1% to the total HIV DNA copy numbers in PBMC of CHI and AHI patients, respectively. Resting CD4 T cells contributed 4.9% to the total HIV DNA copy number in PBMC of CHI patients and 1.9% in AHI patients. None of the comparisons between cell types or type of patients were statistically different.

### Vδ2 cells are a novel reservoir for replication-competent HIV

We performed viral outgrowth assays using highly purified Vδ2 cells to demonstrate the recovery of replication-competent HIV after, but not prior to, activation of Vδ2 cells from HIV-1 infected patients on fully suppressive ART. As expected, percentages of CD3^+^Vδ2 cells were lower in patients treated in CHI compared to patients treated in AHI (mean 0.25% vs. 0.77%; **Table 2**). Replication-competent HIV was detected in at least one culture replicate in 14 out of 18 patients (78%), with no virus recovered in two AHI and two CHI patients. Overall, for AHI patients we assayed 94 cultures of which 21 were positive for HIV p24, compared to 53 total cultures for CHI patients with virus recovered in 20 (22.3% vs. 37.7%; **Table 2**). Therefore, replication-competent virus was more frequently recovered from patients treated in CHI than from AHI-treated patients. In parallel to these assays and as part of other projects in the lab, viral outgrowth assays with isolated r-CD4 cells were performed as previously described [21,23,24]. A summary of the outgrowth assays for r-CD4 cells is included in **Table 2**. Our results show that Vδ2 cells constitute a novel reservoir for HIV infection that may persist for years as latent Vδ2 cell infection was detected in CHI patients despite long-term suppressive ART and the lack of intermittent low-level viremia (“blips”) during the prior two years of clinical follow-up (**Table 1**). In addition, we analyzed two patients (C.2 and C.3) one year after their first evaluation, and replication-competent HIV was again recovered from their Vδ2 cells (S1 Fig). An expanded longitudinal analysis is underway to assess the stability of this reservoir.

**Table 2:** Detection of replication-competent HIV and HIV DNA from isolated Vδ2 cells and resting CD4^+^ T cells (r-CD4).

	Vδ2 cells				r-CD4 cells			
Patient	Vδ2%*	Positive cultures/total	Number of cells cultured (x10^5^)	HIV DNA copies/10^6^ cells	r-CD4%**	Positive cultures/total	Number of cells cultured (x10^6^)	HIV DNA copies/10^6^ cells
**A.1**	1.2	1/15	6.50	2500	12.9	1/24	33.6	n/a
**A.2**	0.9	5/11	3.50	113.2	12.6	5/32	38.8	38.7
**A.3**	0.8	3/11	4.30	n/a	n/a	0/25	8.5	n/a
**A.4**	0.1	0/2	1.60	n/a	n/a	9/19	3.3	n/a
**A.5**	0.1	1/3	0.15	n/a	10.5	12/30	48.6	n/a
**A.6**	n/a	3/14	1.90	n/a	22.8	12/34	30	n/a
**A.7**	0.7	7/22	6.10	1719.5	22.3	10/28	28.8	n/a
**A.8**	1.8	1/10	3.20	1652.1	13.3	18/30	33.8	n/a
**A.9**	0.6	0/6	3.00	<LOQ	12.3	25/36	48.8	25.4
**A.10**	0.5	n/a	n/a	667.25	10.3	n/a	n/a	<LOQ
**Mean**	0.77	21/94	3.00	1108.6	15.3	92/258	30.5	21.3
**C.1**	0.2	1/1	0.80	85.7	18.1	1/36	48.8	4.77
**C.2**	0.4	3/4	0.95	1569.6	13.4	24/30	48.6	n/a
**C.3**	n/a	2/4	0.95	n/a	16.8	4/27	41.1	n/a
**C.4**	n/a	0/1	0.80	n/a	n/a	16/36	12.4	n/a
**C.5**	0.3	0/4	0.95	<LOQ	10.3	9/34	7.3	89.9
**C.6**	0.3	5/6	0.75	81.0	15.6	1/30	19.1	21.1
**C.7**	n/a	6/12	2.35	n/a	14.4	22/30	48.6	n/a
**C.8**	0.1	1/12	1.6	144.2	15.7	18/30	33.8	5.2
**C.9**	0.2	2/9	1.50	n/a	17.5	11/30	33.8	n/a
**C.10**	0.9	n/a	n/a	1951.1	n/a	n/a	n/a	94.5
**Mean**	0.25	20/53	1.18	638.6	15.2	106/283	32.6	35.9

*Percentage of peripheral CD3^+^ Vδ2 cells or **CD3^+^CD4^+^HLA-DR^-^CD69^-^ cells within total PBMC. Antibody cocktail used to isolate r-CD4 cells did not completely deplete γδ T cells.

n/a, not available.

LOQ, limit of quantitation.

**Fig 3:**
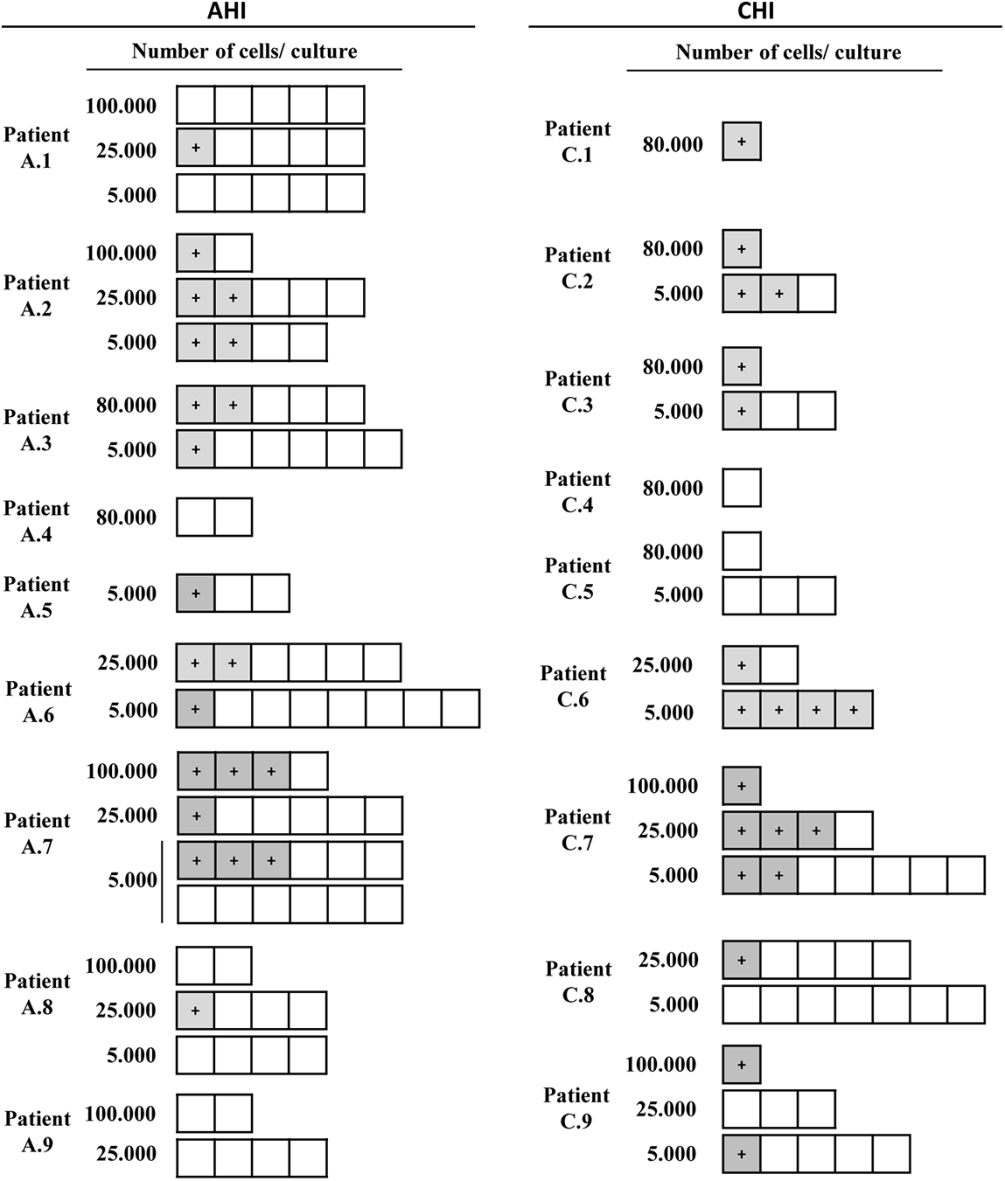
Recovery of replication-competent virus from isolated Vδ2 cells. Representation of the culture conditions in isolated peripheral resting Vδ2 T cells in patients treated in acute HIV infection (AHI) and in chronic HIV infection (CHI). Each square represents one culture replicate that is gray when replication-competent HIV was recovered, and is open when culture replicates were negative. Vδ2 T cells were recovered at a higher frequency in patients treated in AHI than in patients treated in CHI, allowing more replicates for the outgrowth assays. However, HIV was more frequently recovered from CHI patients than from AHI patients.

In ten of the 14 patients in whom virus was recovered (A.2, A.3, A.5, A.6, A.7, C.2, C.3, C.6, C.7 and C.9) HIV was detected in cultures of only 5000 cells, suggesting a high frequency of infection within Vδ2 cells (Fig 3).

**Fig 4:**
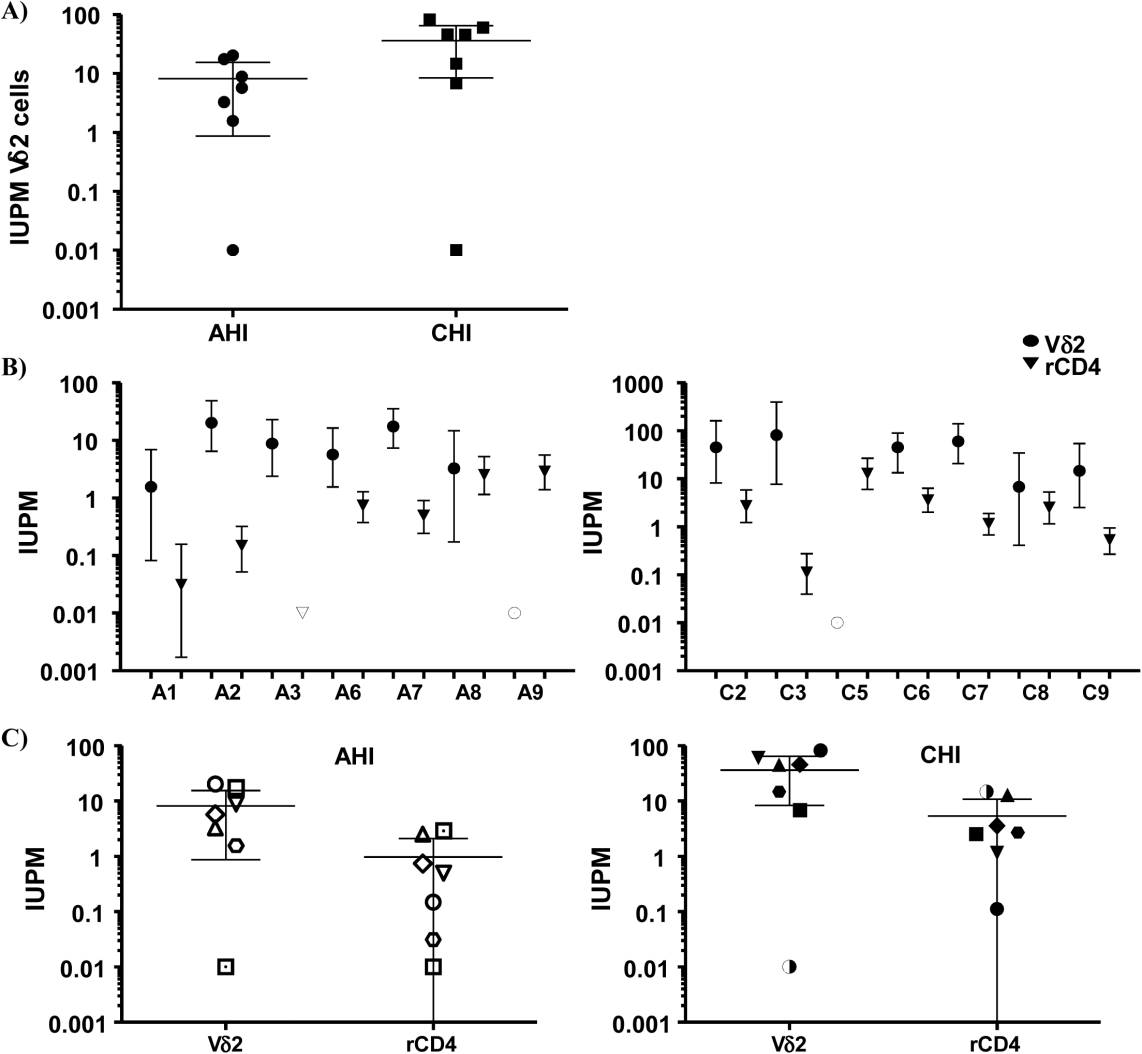
Frequency of replication-competent HIV expressed as infectious units per million (IUPM) cells. **A)** Comparison between the frequency of infection within isolated Vδ2 cells between patients treated in the acute HIV infection (AHI) and patients treated in the chronic HIV infection (CHI). **B)** Point estimate (95% CI) of the frequency of infection in isolated Vδ2 cells (circles) and resting CD4^+^ T (r-CD4) cells (triangles) in individual patients treated in AHI (left panel) or treated in CHI (right panel). Open symbols means HIVp24 was below the limit of detection, that was <10.67 for Vδ2 cells in patient A6, <0.1 for r-CD4 cells in patient A7, and <31.9 for Vδ2 cells in patient C3. **C)** Comparison of the point estimate of the frequency of infection between Vδ2 cells and r-CD4 cells in patients treated in AHI (left panel) and patients treated in CHI (right panel). p> 0.05, Wilcoxon signed-ranked test.

Next, we calculated the frequency of infection expressed as infectious units per million (IUPM) isolated Vδ2 cells in 14 patients with available cell dilutions (seven AHI and seven CHI) (Fig 4A). In addition, IUPM r-CD4 cells from the same patients were calculated to compare both cell populations. Percentages of r-CD4 cells, number of cells cultured and total number of positive and total cells assayed are shown in Table 2. As expected, the confidence interval for Vδ2 cells was much greater than for r-CD4 cells due to the lower number of Vδ2 cells assayed and therefore the estimation of the frequency of infection is less accurate (Fig 4B). However, we could not detect statistical differences when IUPM Vδ2 cells were compared to IUPM r-CD4 cells, suggesting that despite the inaccuracy of the co-culture system, Vδ2 cells may be frequently latently infected.

**Fig 5:**
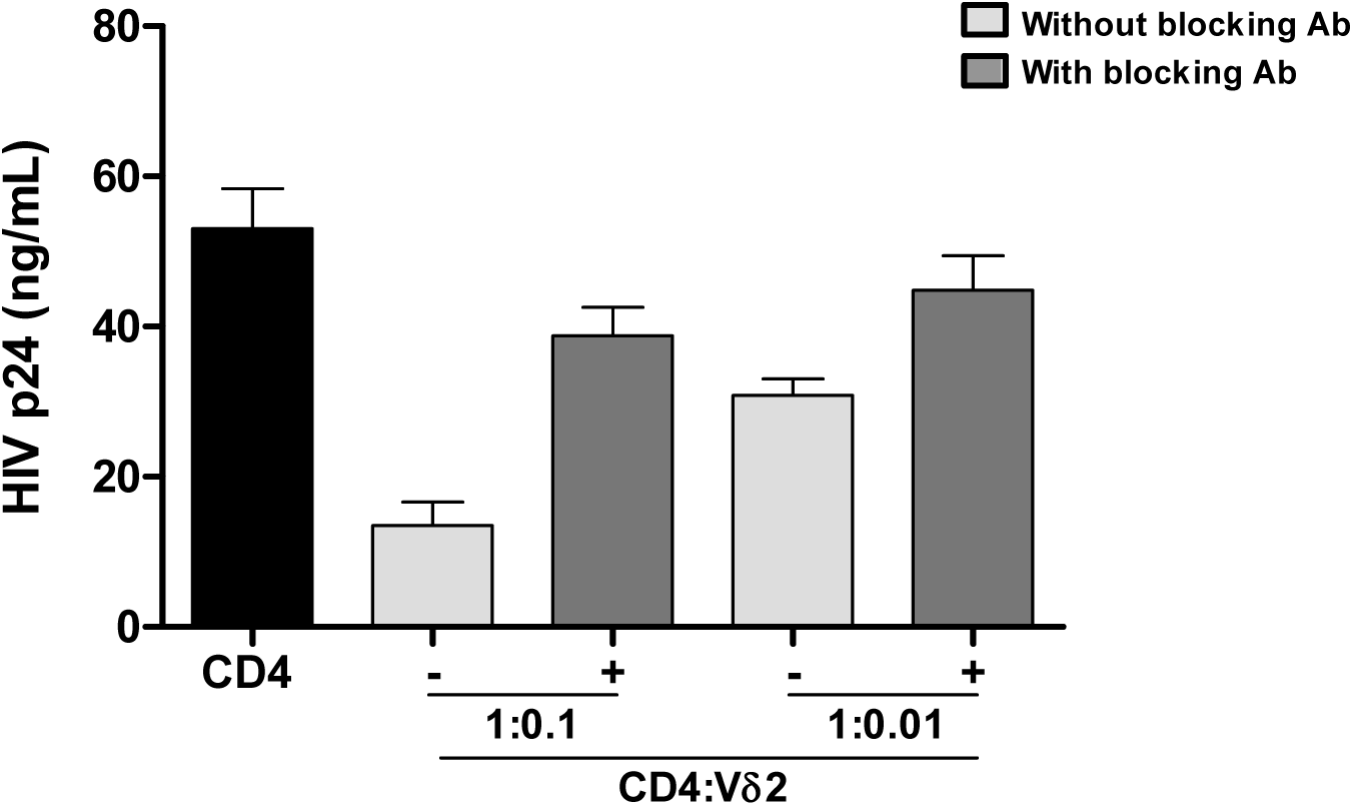
Vδ2 cells exhibit potent anti-HIV activity *in vitro*. Isolated Vδ2 cells from healthy donors were co-cultured with autologous *in vitro* infected HIV- CD4^+^ T cells at 1:0.1 or 1:0.01 ratios (CD4:Vδ2) for seven days. HIV p24 production was inhibited in a cell-dose-dependent manner. When a cocktail of blocking antibodies against CD8, CD16 and NKG2D were added, cytotoxic function of Vδ2 cells was partially abrogated. Mean±SE of three different donors is represented.

Interestingly, while quantifying the frequency of infection within Vδ2 cells by limiting dilution assay, in some patients we found an unusual pattern of recovery of HIV with more positive wells at lower dilutions and no virus recovered when more cells were cultured (Fig 3). As γδ T cells possess innate, nonspecific antiviral function [25,26], we hypothesized that an antiviral activity of the uninfected γδ T cells might reduce the recovery of HIV in cultures with high cell inputs, yielding the unusual pattern of viral outgrowth seen in some patients. To test this hypothesis, we performed viral inhibition assays, co-culturing Vδ2 cells from a healthy, uninfected donor, with autologous CD4^+^ T cells that had been HIV-infected *ex vivo* at ratios of 1 CD4 cell and 0.1 or 0.01 γδ cells. Vδ2 T cells inhibited HIV production from infected CD4^+^ T cells, with increased inhibition seen at higher cell inputs in the co-culture system (Fig 5). In addition, in some wells we blocked the cytotoxic activity of γδ T cells by pre-incubating the isolated Vδ2 cells with a cocktail of antibodies against CD8, NKG2D and CD16 (Fig 5). HIV p24 production was 74.6% inhibited in the 1:0.1 ratio conditions and 41.8% in the 1:0.01 conditions. When the cocktail of antibodies was used, these percentages decreased to 26.9% and 15.5% in the 1:0.1 and 1:0.01 ratios, respectively.

### Mechanism of HIV infection

**Fig 6:**
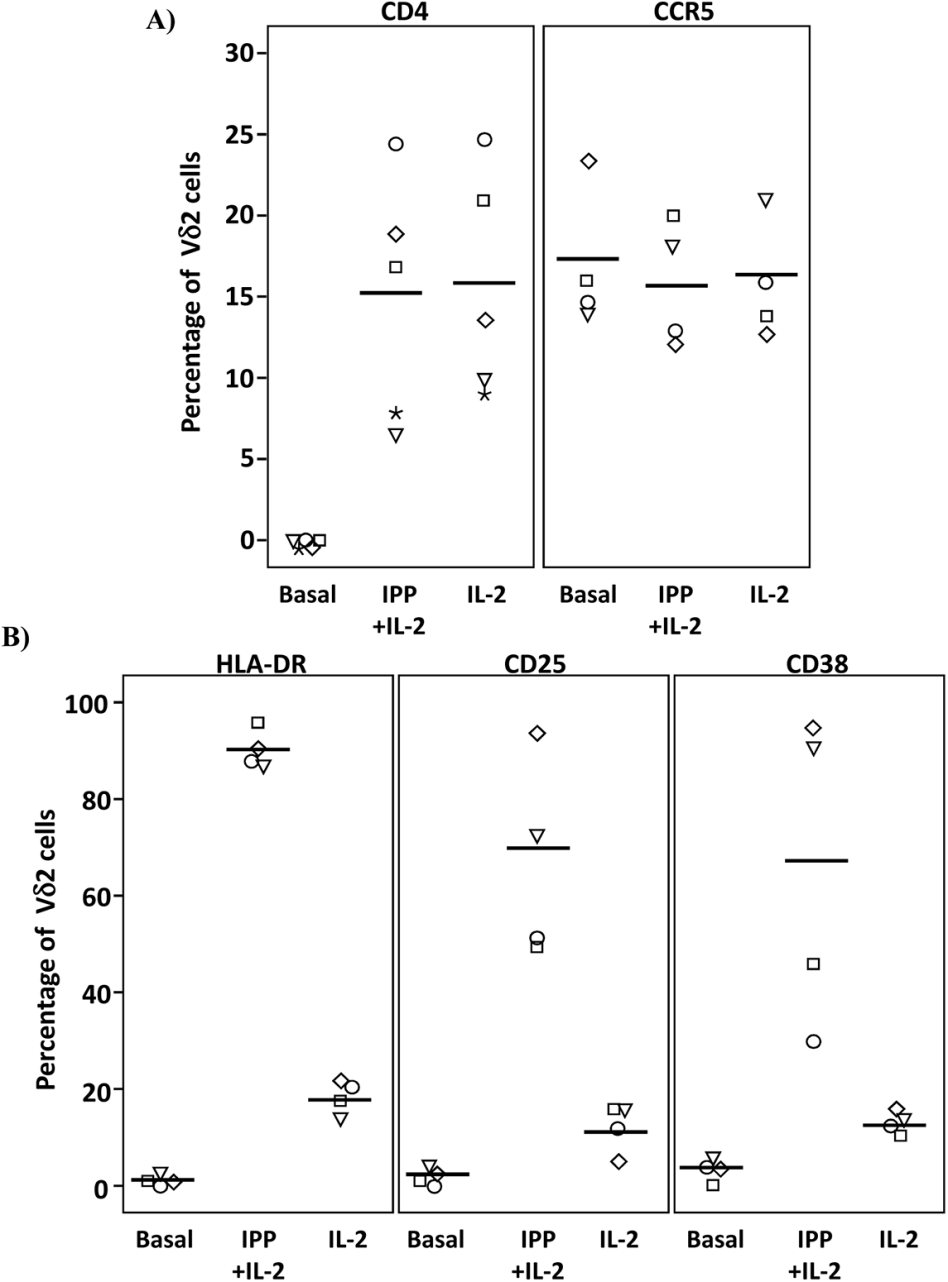
Expression of CD4, CCR5 and activation markers on Vδ2 cells. PBMC from HIV-uninfected donors were cultured and treated with IL-2 alone or IPP and IL-2 for six days. The surface expression of CD4, CCR5, and activation markers (HLA-DR, CD38 and CD25) on Vδ2 cells were analyzed by flow cytometry on days 0 and 6. **A)** Majority of peripheral Vδ2 cells do not express the CD4 receptor but CD4 is significantly upregulated after six days in culture with IL-2 or with IPP and IL-2 (*p<0.01). However, CCR5 is expressed on the surface of Vδ2 cells at baseline without a significant induction after treatments. **B)** Analysis of activation markers (HLA-DR, CD25 and CD38) showed peak activation after six days of culture with IPP and IL-2 with moderate activation by IL-2 alone (*p<0.05).

Isolated Vδ2 cells can be infected *in vitro*, [17,18] (S2 Fig) despite low or absent surface CD4 receptor expression prior to activation and HIV infection of these cells is inhibited by CD4 blockade [17], (S2 Fig). We further investigated the CD4-dependence of HIV infection in Vδ2 cells. Total PBMC from uninfected donors were activated with IL-2 alone or IPP and IL-2, and surface marker expression was analyzed by flow cytometry (Fig 6A and S3 Fig). As expected, CD4 receptor expression was detected on <0.3% of Vδ2 cells at day 0, but became detectable on up to 25% of cells after six days of culture in the presence of IL-2 alone, or IPP and IL-2. All increases were statistically significant (p <0.01; Fig 6A). Interestingly, treatment with exogenous IL-2 alone induced CD4 expression to similar levels as did IPP and IL-2 (mean 15.3% and 15.9%, respectively). In contrast, none of these conditions significantly increased the surface expression of CCR5 after six days in culture (Fig 6A). In addition, the activation status of Vδ2 cells was also assessed in the same cells by analyzing the expression of the MHC Class II HLA-DR receptor, the IL2 receptor alpha chain CD25, and the activation marker CD38 (Fig 6B). After six days in culture with IL-2 alone or IPP and IL-2 the expression of these markers was significantly increased (p <0.05 in all cases) although treatment with IL-2 alone induced activation in no more than 20% of Vδ2 cells (Fig 6B).

**Fig 7:**
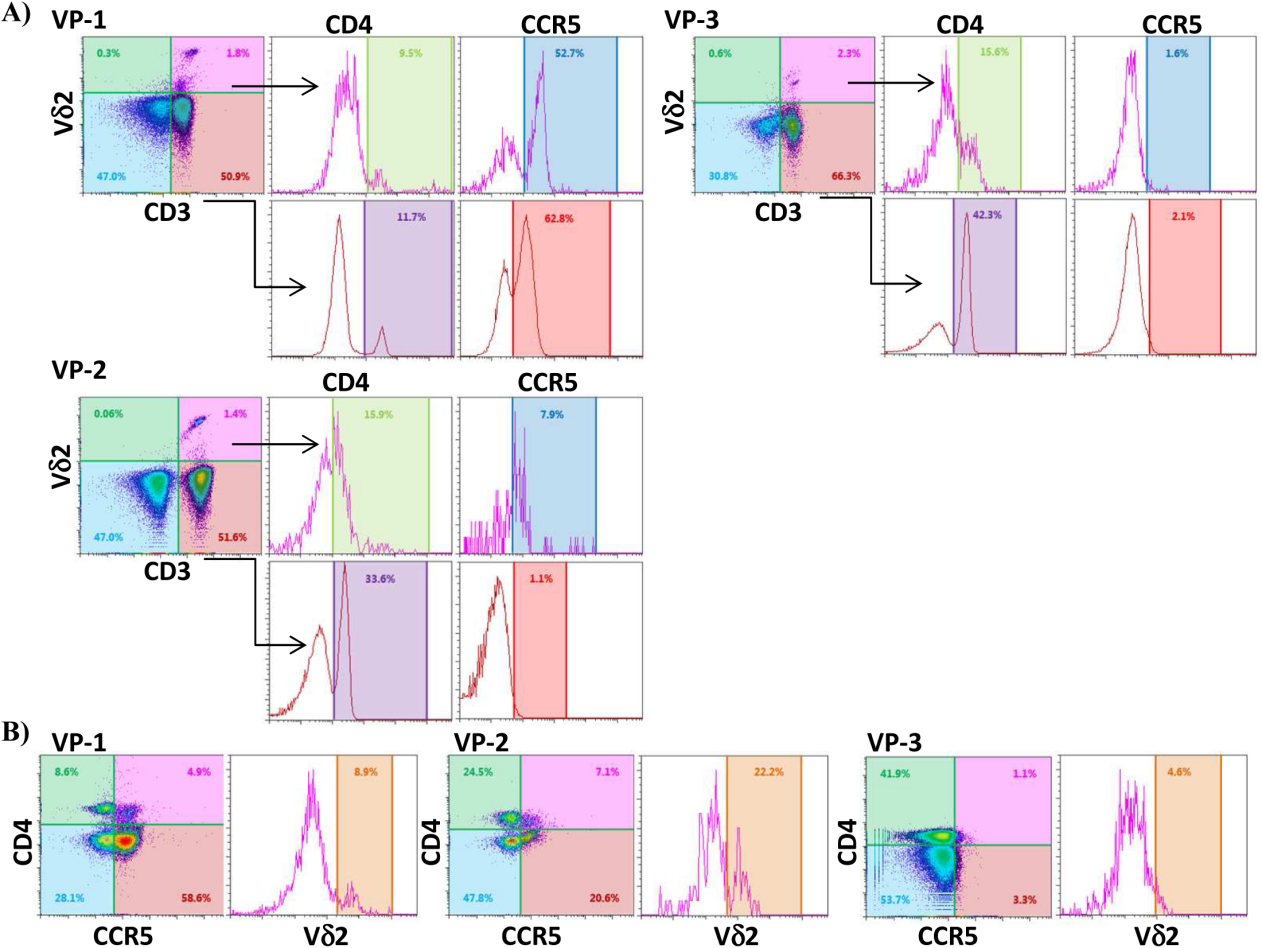
Vδ2 cells from untreated recently HIV-infected patients express CD4 and CCR5. **A)** Dot plots from three recently infected patient (VP1, VP2 and VP3) showing surface expression of CD4 and CCR5 on CD3^+^Vδ2^+^ cells, and on CD3^+^Vδ2^-^ cells for comparison. **B)** Density plots showing the percentage of Vδ2 cells coexpressing CD4 and CCR5 in the same three patients.

Based on these results we hypothesized that immune activation, driven in this instance by HIV infection, might upregulate CD4 expression on Vδ2 cells *in vivo*. To test this hypothesis we measured surface expression of CD4 and CCR5 expression in Vδ2 cells donated by three viremic patients diagnosed during the acute phase of HIV infection, prior to the initiation of ART (Fig 7). Based on history and diagnostic testing, the estimated date of infection in these patients was less than 23 days prior to sampling. Likely related to the pathological immune activation of acute HIV infection, 9.5%, 15.6% and 15.9% of Vδ2 cells expressed CD4 (Fig 7A), as compared to <0.3% of Vδ2 cells in healthy donors. In addition, we also analyzed the percentage of Vδ2 cells that coexpressed CD4 and CCR5 (Fig 7B). This observation in these unique patients supports our hypothesis that pathological immune activation in early HIV infection promotes the upregulation of CD4 expression in Vδ2 cells, making them targets for HIV infection *in vivo*.

## Discussion

We have discovered that peripheral resting Vγ9Vδ2 cells can act as a cellular reservoir of persistent, latent HIV infection. Using a gold-standard coculture assay that defines the presence of latent infection, we find that the frequency of replication-competent virus in these cells is substantial, with virus recovered in as few as 5000 Vδ2 T cells that lack activation markers. This unexpected finding is supported by the detection of HIV DNA within this cell population, implying that at least a fraction of the HIV DNA detected within Vδ2 cells represents replication-competent virus.

We propose a mechanism to explain the infection of Vδ2 cells despite the absence of CD4 expression in their surface. As isolated Vδ2 cells can be infected *in vitro* we hypothesize that the activated immune environment during untreated HIV infection induces transient CD4 upregulation, rendering Vδ2 cells permissive for HIV-1 infection, and founding a substantial population of latently infected γδ cells. This hypothesis is supported *in vivo* by the detection of expression of CD4 and CCR5 in Vδ2 cells in untreated patients in early acute HIV infection. Latent infection of γδ cells could occur at other times as well, when cellular activation results in upregulation of CD4 expression.

Recovery of purified Vδ2 cells was reduced in patients treated in CHI, compared to patients treated in AHI, as there is a dramatic loss of Vδ2 cells early after infection with HIV [14,15,18]. Such depletion is only partially reversed by ART [27]. Remarkably, replication-competent HIV was quantified in purified Vδ2 cells in 77% of patients who had been treated and suppressed for a median of nearly four years. Although γδ T cells represent only a small fraction of the total CD3^+^ T lymphocytes, frequency of infection within isolated Vδ2 cells was not statistically different from that in r-CD4 cells. However, it is important to highlight that this estimation is not as accurate as the estimation for r-CD4 cells, as reflected by the 95% confidence intervals, because a significantly lower number of cells were assayed. It is also important to note that maximum activation conditions were differently assayed for both cell populations. Our results may overestimate the frequency of infection in γδ cells compared to r-CD4 cell calculations, as viral outgrowth starts during the first 24 hours of incubation, when γδ cells can spread the virus to the γδ-CD8-depleted-PBMC, required for Vδ2 cell activation. In addition, our data suggest the possibility that the ratio of defective to replication-competent proviruses might be lower in Vδ2 cells although future comprehensive sequence analysis will be required to fully address this possibility. A relative deficiency of restriction factors such as APOBEC3G/3F [28,29] in γδ T cells might explain these findings. In addition, a recent study has shown that HIV DNA might be present in cells other than conventional CD4^+^ T cells and myeloid cells suggesting that HIV may persist in other cell types [30]. As γδ cells can be phenotypically classified using CD45RA and CD27 markers, it is possible that in some prior studies these cells may have been included in the evaluation of HIV infection within the latently infected resting memory cell population. Interestingly, our results and others [22] have shown that in some patients, HIV DNA levels within r-CD4 cells are lower than in total PBMC, suggesting that in those patients, other cell types might have a high contribution to total HIV DNA measurements. In addition, activated T cells have also been reported to have a greater HIV DNA contribution than r-CD4 cells [23,24]. Although we found higher levels of HIV DNA within isolated γδ T cells, the rarity of these cells within PBMC makes them to less frequent contributors to the total reservoir than r-CD4 cells. Of note, the custom antibody cocktail used to isolate r-CD4 cells does not completely deplete the γδ T cells and therefore comparisons between r-CD4 cells and γδ T cells are not totally accurate. We have begun studies to evaluate the exact contribution of γδ T cells in assays using total r-CD4 cells.

Although we recovered HIV from low numbers of γδ T cells in 14 of 18 patients, in several patients the recovery of replication-competent virus was less than expected in cultures with higher Vδ2 cell input. In these cases, estimates of the frequency of infection cannot be made with these data as such estimates depend on the assumption of a normal distribution of infection. We speculate that in these cultures, sufficient Vδ2 cells were present to exert potent antiviral activity [25,26], leading to inhibition of spread of the virus during the outgrowth phase of the assay. In support of this, we demonstrate a dose-dependent inhibition of the p24 production in culture when isolated *in vitro*-HIV-infected CD4 cells were cocultured with Vδ2 cells. We also show that blocking CD16, NKG2D and CD8 receptors, Vδ2 cell cytotoxic capacity is partially inhibited suggesting that other receptors are also involved in exerting this function.

HIV can infect isolated γδ T cells *in vitro* [17,18]. In this study, we have shown that this occurs through a mechanism that involves the transient upregulation of the CD4 receptor after activation. CD4 expression is upregulated in Vδ2 cells *in vitro* following activation with IPP and IL-2. This transient upregulation of CD4 has also been reported after infection of γδ T cells with human herpesvirus [31]. Treatment with HMBPP and IL-2 also induced the expression of CD4. Although the vast majority of Vδ2 lymphocytes do not express CD4, in the setting of lymphopenia, rapid T-cell turnover, or heightened immune activation, increased IL-2 levels could lead to CD4 upregulation in Vδ2 cells, making them susceptible to HIV infection *in vivo*. We directly observed this phenomenon prior to ART in viremic, newly infected patients, suggesting that this mechanism is plausible *in vivo*. Therefore, in addition to a previously reported indirect mechanism to explain peripheral Vδ2 cell depletion [16], we describe a potential direct effect of HIV infection on Vδ2 cell depletion. Direct infection of Vδ2 cells by HIV could lead to depletion of most Vδ2, while others might survive and establish latent infection. Infection of Vδ2 cells can have significant consequences, as these cells constitute an important bridge between the innate and adaptive immune response [32]. Therefore, due to reduced Vδ2 cell signaling, dendritic cell function [33–36], follicular B cell help, or CD4^+^ T cell responses [37] might be impaired.

In summary, our results demonstrate that Vδ2 cells are a novel latent reservoir for replication-competent HIV. Although these T lymphocytes are generally rare, the frequency of latent infection in these cells makes it likely that they contribute measurably to the total burden of latent, quiescent HIV infection. Moreover, we demonstrate that isolated Vδ2 cells can be infected *in vitro*, and illustrate a mechanism that could allow γδ T cell infection despite constitutive low expression of the CD4 receptor. We found that activation of Vδ2 cells upregulates CD4 expression, enabling HIV infection. The durability of latent infection within this novel cell population must still be established in longitudinal studies. However, given the broad efforts to discover reagents that disrupt latency in resting CD4 central memory cells as a first step towards viral eradication therapies, it may be necessary to also address the responsiveness of proviral HIV genomes within resting γδ T cells to such strategies.

## Materials and Methods

### Patients and healthy donor volunteers

All patients provided written informed consent, and studies were approved by the UNC Institutional Review Board. Our criteria to define and select patients treated in acute HIV infection (AHI) have previously been reported [38,39]. Briefly, patients identified in AHI (plasma HIV RNA positive and HIV Western blot negative) were enrolled and initiated ART within 45 days of the estimated date of infection. Serial measurements of plasma viremia and CD4^+^ T cell count were performed, and when patients were aviremic (<50 HIV RNA copies/ml) on ART for >6 months, cells were obtained by continuous-flow leukapheresis. Patients studied after initiation of ART in chronic HIV infection (CHI) had a history of stable, successful treatment, and plasma HIV-1 RNA levels <50 copies/mL for >2 years without blips. Buffy coats from uninfected donor volunteers were obtained from the New York Blood Center (Long Island City, NY, USA).

### High purity peripheral resting γδ T cell isolation

As part of the routine preparation for the outgrowth assay, prior to γδ T cell isolation, freshly isolated patients’ PBMC were incubated with raltegravir and abacavir for 24 hours to avoid any potential *de novo* infection due to HIV reactivation. For infectivity assays, isolated γδ T cells from fresh PBMC from healthy non-HIV infected were used. γδ T cells were first enriched by negative selection using a commercially available cocktail containing monoclonal antibodies (mAb) directed against non-γδ cells, including antibodies against granulocytes, red blood cells, dendritic cells, pan-αβ T cells, NK cells, stem cells, monocytes (StemCell Technologies, Vancouver, Canada) and afterwards the cells were isolated by fluorescent activated cell sorting (FACS) using a Reflection sorter (iCyt, Champagne, IL, USA) or a FACSAria II (BD). Each sorting experiment was validated performing instrument quality controls, and running isotype controls and fluorescence minus one control. In addition, gating strategy for each specific experiment was based on the selection of the target cell by CD3 (clone SK7, BD) and Vδ2 (clone B6, Biolegend, San Diego, CA) mAbs, and exclusion of potential contamination of CD4^+^ T cells in a third channel using the CD4 mAb (clone RPA-T4, BD), and in some experiments αβTCR mAb (clone MOPC-21, Biolegend). To identify singlets we performed a 2-step doublet discrimination using HxW of the pulse; first in the SSC direction then in the FSC direction. In all our preparations the singlets were always well separated from the doublets and no contamination with αβ T cells or CD4^+^ T cells was detected in the presort sample (Fig 1). In addition, HLA-DR mAb (clone TU36, BD) was used to exclude potential pre-activated γδ T cells, and fixable aqua (Life Technologies, Grand Island, NY) was used to exclude dead cells. Cells were collected in RPMI-1640 containing stable Glutamine and Hepes (Gibco, Life Technologies, Grand Island, NY), 10% pooled human AB serum (Sigma-Aldrich, St Louis, MO) and 10% PenStrep (Sigma-Aldrich) (hereafter referred to as γδ medium). After isolation, an aliquot was used to analyze the purity of the sorted population (> 99% γδ T cells, < 1% all other cells) (Fig 1).

### HIV outgrowth assays

After sorting, Vδ2 T cells were centrifuged and resuspended in the suitable volume of γδ medium to perform the standardized co-culture assay [20,21]. As part of the standard method, cells were plated in limiting dilution, when possible, and activated with 100nM HMBPP (kindly provided by Dr. H. Jomaa, Justus-Liebig University, Giessen, Germany) or 1μM IPP (Sigma) and 100U/mL IL-2 (Peprotech, Rocky Hill, CT) for 24 hours. In addition, as γδ T cell activation requires the presence of APC [40] but γδ T cells and CD8^+^ T cells possess anti-HIV activity [26], allogeneic non-HIV infected PBMC used as APC were first depleted of γδ T cells and CD8^+^ T cells, added to cultures at a 1:4 ratio (isolated γδ T cells: PBMC depleted), and incubated for 24 hours at 37°C and 5% CO_2_. Vδ2 cells were then washed and resuspended in γδ medium containing 10U/mL IL-2 and cocultured with allogeneic PHA-activated PBMC depleted of CD8^+^ T cells as target cells. CD8^+^ T cells were depleted by negative magnetic isolation following manufacturer’s instructions (StemCell Technologies). Complete medium containing 10U/mL IL-2 was replaced and refreshed every three or four days. Supernatants were stored at -80°C for analysis of viral p24 production by ELISA (ABL_inc_, Rockville, MA, USA) at days 15, and 19. As a control Vδ2 cells were cultured without activation prior to the addition of target cells in 5U/mL IL-2, following the same coculture protocol, which were uniformly negative. Quantitative viral outgrowth assay for r-CD4 cells was performed in parallel using the standard procedure previously reported in our lab [21,23,24] and described above also for γδ cells. However, activation procedures were different between both cell populations. Briefly, magnetically isolated r-CD4 cells were activated with 2μg/mL PHA, 60U/mL IL-2 and irradiated PBMC from a non-infected donor, and target cells were added as previously reported and as explained above. Infectious units per million Vδ2 and r-CD4 cells (IUPM) were calculated using the R software developed at the University of North Carolina that allows calculating the point estimate of the frequency of infection and the 95% confidence interval.

### HIV DNA quantification

Total HIV *pol* DNA copies within isolated Vδ2 cells, unfractionated PBMC and resting CD4^+^ T cells were quantified by droplet digital PCR (ddPCR) using primers, probes and conditions previously reported [41]. Briefly, DNA was extracted from frozen cell pellets of 1x10^5^ Vδ2 cells and 1x10^6^ PBMC and r-CD4 cells on average, using the Qiagen DNeasy Blood and Tissue kit (Qiagen, Maryland, USA) and concentration of DNA was measured using the Nanodrop (Thermo Scientific). PCR reactions were loaded into the Bio-Rad QX-100 droplet generator. Each reaction consisted of a 20μL mix containing 10 μL ddPCR Probe Supermix, 900 nM primers, 250 nM probe, and template DNA. Following amplification in a standard thermo cycler (10 min. at 95°C, 40 cycles of 30 sec. at 94°C, 60 sec. at 58°C and final 10 min. at 98°C) droplets were immediately analyzed as positive or negative using the Bio-Rad QX-100 droplet reader. The no template controls were used to set the threshold, and copy number was calculated using the manufacturer’s software and normalized to the total number of cells (Bio-Rad QuantaSoft v.1.2). As a control, dilutions of DNA from J89 cells, which contain a single copy of HIV, was used in all runs. In addition, to control for the different number of cells assayed, we run independent assays to compare HIV DNA levels in PBMC samples of 1x10^5^ cells and 1x10^6^ cells, which showed similar copy number.Samples were run in triplicate and the limit of quantitation (LOQ) was calculated based upon the frequency of false positives in the no template control.

### Calculations of the contribution of HIV DNA to the HIV reservoir

We calculated the contribution of HIV DNA in Vδ2 cells and r-CD4 cells to the total HIV-DNA^+^ PBMC as follows: First, we calculated the total HIV DNA copies in each cell population by multiplying the average HIV copy number per million cells to the percentage of Vδ2 or r-CD4 cells present in total PBMC. Then, this total HIV copy number was divided by the total HIV copy number in PBMC to obtain the proportion of HIV corresponding to Vδ2or r-CD4 populations. Vδ2 cells contributed 1.6% and 8.1% to the total HIV DNA copy numbers in PBMC of CHI and AHI patients, respectively. Resting CD4 T cells contributed 4.9% to the total HIV DNA copy number in PBMC of CHI patients and 1.9% in AHI patients. None of the comparisons between cell types or type of patients were statistically different.

### Vδ2 cell HIV-infection assays

Freshly isolated Vδ2 cells from HIV-negative volunteers were activated using 100nM HMBPP and 100U/mL IL-2 for 24 hours and spinoculated for 2 hours with 1ng/mL of the CCR5 JR-CSF strain. Cells were then washed twice to remove the excess of virus, cultured in triplicate in γδ medium containing 20U/mL of IL-2 for 7 days and supernatants were analyzed for HIV p24 production on days 4 and 7. As a control for the infection conditions, isolated CD4^+^ T cells from the same uninfected blood donor volunteer were stimulated in parallel using 2μg/mL PHA and 60U/mL IL-2. Supernatants from day 0 (basal levels after virus exposure) were also stored and measured as a negative control. In some wells, Vδ2 cells and CD4 cells were incubated with 50μg/mL of an anti-CD4 mAb (clone RPA-T4, BD) before exposure to HIV. Experiment was repeated using three different donors.

### HIV inhibition assays

Vδ2 and CD4^+^ T cells were FACS-sorted from PBMC of healthy donor volunteers. CD4 cells were activated with 2μg/mL PHA and 60U/mL IL-2 for 24h washed and infected by spinoculation with the viral strain JR-CSF following the same protocol described above, and co-cultured in triplicate at different ratios of autologous Vδ2 cells (1:0.1 and 1:0.01 CD4:Vδ2). In some wells cytotoxic activity of γδ cells was blocked using a mixture of purified mAb against CD8 and CD16 from Biolegend, and NKG2D from Miltenyi Biotec. Media was refreshed at days 4 and 7 and supernatants stored until p24 ELISA quantification (ABL_inc_, Rockville, MA, USA). Experiments were performed in three different donors.

### Surface marker expression on Vδ2 cells

PBMC from healthy donor volunteers isolated from fresh buffy coats were incubated with 1μM IPP and 100U/mL IL-2 or 100U/mL IL-2 alone. Expression of CD4 and CCR5 receptors along with expression of activation markers HLA-DR, CD25 and CD38, was controlled by flow cytometry at days 0 and six of culture. mAb used were; CD4-FITC (clone RPTA-4, BD), Vδ2-PE (clone B6, Biolegend), CCR5-V450 (clone 2D7/CCR5, BD), HLA-DR-FITC (clone TU36, BD), CD25-V450 (clone M-A251, BD) and CD38-PerCPCy5.5 (clone HIT2, BD). Briefly, cells were blocked with FBS (Sigma) for 10 minutes on ice, resuspended in staining buffer (PBS-2% FBS), incubated for 20 minutes on ice in the dark with combinations of the mAb, or suitable isotype controls, and washed twice. Cells were then fixed with 2% paraformaldehyde solution and analyzed in the Attune acoustic cytometer (Applied Biosystems).

### Statistical analyses

Differences between patients treated in AHI and patients treated in CHI were compared using the two-tailed Mann-Whitney *U*-test and comparisons between mean values were performed by the two-tailed Student t-test. Statistical Analyses were performed using the IBM-SPSS version 21.0 (Chicago, Illinois, USA) and p values <0.05 were considered statistically significant.

## Supporting Information

S1 Fig Recovery of replication-competent virus from the follow-up of two CHI-treated patients. HIV was recovered in patients C.2 and C.3 when measured a second time after an additional year of suppressive ART (sustained plasma viral load<50 copies/mL). Each square represents one culture replicate that is represented in gray when replication-competent HIV was recovered, and open squares represent culture replicates negative for HIV p24.
(TIF)

S2 Fig Infection of isolated Vδ2 cells. Purified Vδ2 cells or CD4^+^ T cells from the same HIV-uninfected donor were exposed to HIV JR-CSF. Vδ2 cells were activated prior to exposure to HIV with HMBPP and IL-2. As a comparator, isolated CD4^+^ T cells were also infected following PHA and IL-2 activation. Cells were successfully infected as demonstrated by production of HIV p24 antigen after seven days of culture. Preincubation with anti-CD4 mAb prior to exposure to HIV inhibited viral production, showing that infection was CD4-dependent.
(TIF)

S3 Fig CD4 and CCR5 expression on Vδ2 cells. Flow cytometry analysis of CD4 and CCR5 expression on Vδ2 cells of a representative donor. Contour plots show the percentage of peripheral Vδ2 cells comparing treatment with IL-2 alone IPP and IL-2 and IPP alone. Vδ2 cells expanded, while IL-2 alone did not induce an expansion of the cells. Contour plots in the middle show CD4 and CCR5 expression on Vδ2 cells and lower plots show isotype controls.
(TIF)
