# Supplementary figures and images for: Peripheral Vγ9Vδ2 T Cells Are a Novel Reservoir of Latent HIV Infection
Source: PLoS Pathog. 2015 Oct 16;11(10):e1005201. doi: 10.1371/journal.ppat.1005201 (PMC4608739; doi:10.1371/journal.ppat.1005201)

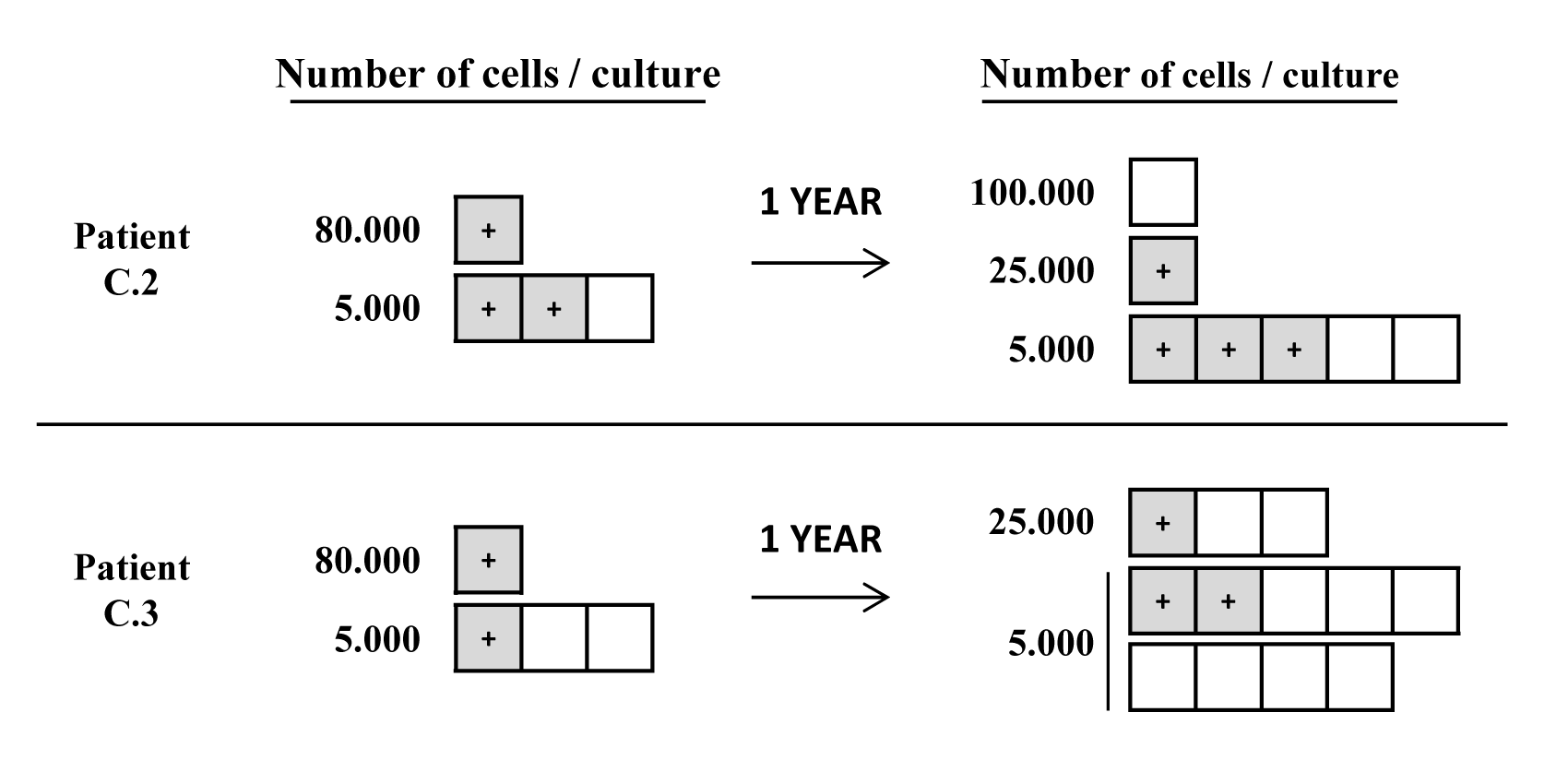

Supplement: S1 Fig — HIV was recovered in patients C.2 and C.3 when measured a second time after an additional year of suppressive ART (sustained plasma viral load<50 copies/mL). Each square represents one culture replicate that is represented in gray when replication-competent HIV was recovered, and open squares represent culture replicates negative for HIV p24. (TIF) [file ppat.1005201.s001.tif]

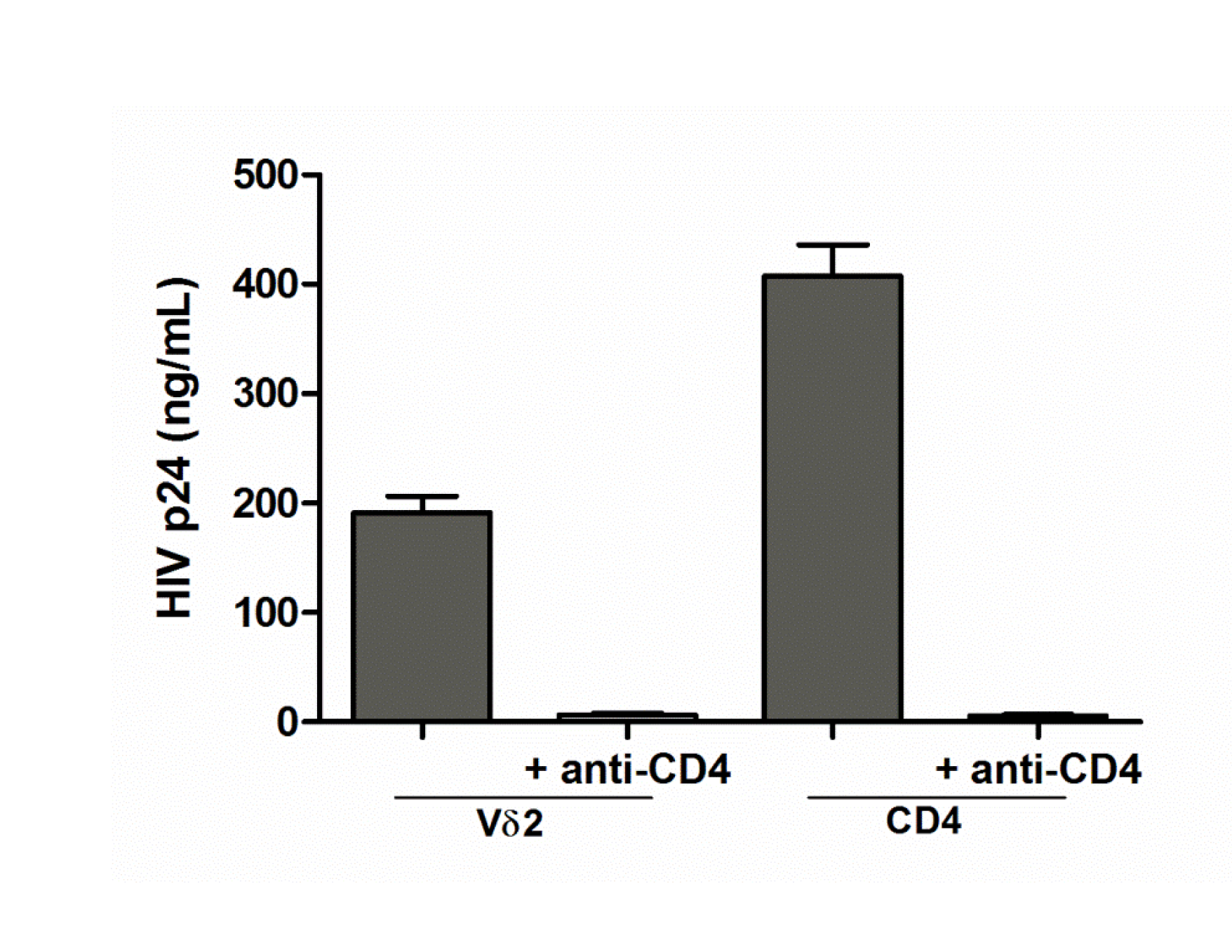

Supplement: S2 Fig — Purified Vδ2 cells or CD4+ T cells from the same HIV-uninfected donor were exposed to HIV JR-CSF. Vδ2 cells were activated prior to exposure to HIV with HMBPP and IL-2. As a comparator, isolated CD4+ T cells were also infected following PHA and IL-2 activation. Cells were successfully infected as demonstrated by production of HIV p24 antigen after seven days of culture. Preincubation with anti-CD4 mAb prior to exposure to HIV inhibited viral production, showing that infection was CD4-dependent. (TIF) [file ppat.1005201.s002.tif]

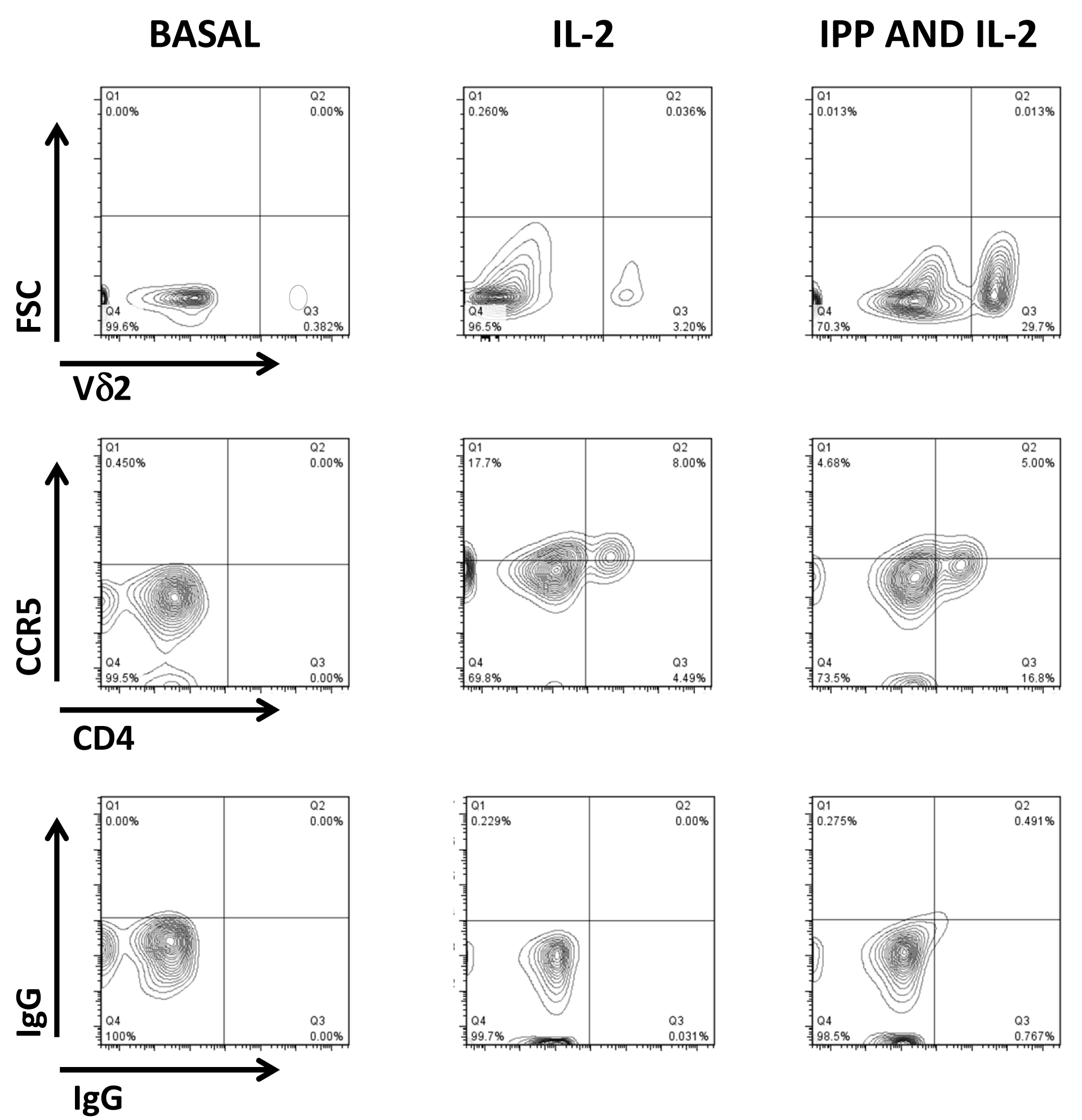

Supplement: S3 Fig — Flow cytometry analysis of CD4 and CCR5 expression on Vδ2 cells of a representative donor. Contour plots show the percentage of peripheral Vδ2 cells comparing treatment with IL-2 alone IPP and IL-2 and IPP alone. Vδ2 cells expanded, while IL-2 alone did not induce an expansion of the cells. Contour plots in the middle show CD4 and CCR5 expression on Vδ2 cells and lower plots show isotype controls. (TIF) [file ppat.1005201.s003.tif]
